# Supplementary material for: Improving the Efficacy of Mesenchymal Stem/Stromal-Based Therapy for Treatment of Inflammatory Bowel Diseases
Source: Biomedicines. 2021 Oct 20;9(11):1507. doi: 10.3390/biomedicines9111507 (PMC8615066; doi:10.3390/biomedicines9111507)
Supplement: Supplementary file 1 [file biomedicines-09-01507-s001.zip › biomedicines-1399337-suppl 2/Supplementary References_proofreading.pdf]

Supplementary References

142. Tanaka F, Tominaga K, Ochi M, Tanigawa T, Watanabe T, Fujiwara Y, Ohta K, Oshitani N, Higuchi K, Arakawa T. Exogenous administration of mesenchymal stem cells ameliorates dextran sulfate sodium-induced colitis via anti-inflammatory action in damaged tissue in rats. *Life Sci* (2008) doi:10.1016/j.lfs.2008.09.016
143. Yabana T, Arimura Y, Tanaka H, Goto A, Hosokawa M, Nagaishi K, Yamashita K, Yamamoto H, Adachi Y, Sasaki Y, et al. Enhancing epithelial engraftment of rat mesenchymal stem cells restores epithelial barrier integrity. *J Pathol* (2009) doi:10.1002/path.2535
144. Zhang Q, Shi S, Liu Y, Uyanne J, Shi Y, Shi S, Le AD. Mesenchymal Stem Cells Derived from Human Gingiva Are Capable of Immunomodulatory Functions and Ameliorate Inflammation-Related Tissue Destruction in Experimental Colitis. *J Immunol* (2009) doi:10.4049/jimmunol.0902318
145. Tanaka H, Arimura Y, Yabana T, Goto A, Hosokawa M, Nagaishi K, Yamashita K, Yamamoto H, Sasaki Y, Fujimiya M, et al. Myogenic lineage differentiated mesenchymal stem cells enhance recovery from dextran sulfate sodium-induced colitis in the rat. *J Gastroenterol* (2011) doi:10.1007/s00535-010-0320-7
146. He XW, He XS, Lian L, Wu XJ, Lan P. Systemic infusion of bone marrow-derived mesenchymal stem cells for treatment of experimental colitis in mice. *Dig Dis Sci* (2012) doi:10.1007/s10620-012-2290-5
147. Li L, Liu S, Xu Y, Zhang A, Jiang J, Tan W, Xing J, Feng G, Liu H, Huo F, et al. Human umbilical cord-derived mesenchymal stem cells downregulate inflammatory responses by shifting the Treg/Th17 profile in experimental colitis. *Pharmacology* (2014) doi:10.1159/000354883
148. Xu X, Chen C, Akiyama K, Chai Y, Le AD, Wang Z, Shi S. Gingivae contain neural-crest- and mesoderm-derived mesenchymal stem cells. *J Dent Res* (2013) doi:10.1177/0022034513497961
149. Abdel Salam AG, Ata HM, Salman TM, Rashed LA, Sabry D, Schaalan MF. Potential therapeutic utility of mesenchymal stem cells in inflammatory bowel disease in mice. *Int Immunopharmacol* (2014) doi:10.1016/j.intimp.2014.07.030
150. Gonçalves F da C, Schneider N, Pinto FO tesbelgu., Meyer FS, Visioli F, Pfaffenseller B, Lopez PL da C, Passos EP andolf., Cirne-Lima EO bin., Meurer L, et al. Intravenous vs intraperitoneal mesenchymal stem cells administration: what is the best route for treating experimental colitis? *World J Gastroenterol* (2014) doi:10.3748/wjg.v20.i48.18228
151. Gonzalez-Rey E, Delgado M. Therapeutic application of mesenchymal stromal cells in murine models of inflammatory bowel disease. *Methods Mol Biol* (2014) doi:10.1007/978-1-4939-1453-1_27
152. Lv Y, Xu X, Zhang B, Zhou G, Li H, Du C, Han H, Wang H. Endometrial regenerative cells as a novel cell therapy attenuate experimental colitis in mice. *J Transl Med* (2014) doi:10.1186/s12967-014-0344-5
153. Banerjee A, Bizzaro D, Burra P, Di Liddo R, Pathak S, Arcidiacono D, Cappon A, Bo P, Conconi MT, Crescenzi M, et al. Umbilical cord mesenchymal stem cells modulate dextran sulfate sodium induced acute colitis in immunodeficient mice. *Stem Cell Res Ther* (2015) doi:10.1186/s13287-015-0073-6
154. Li M, Zhang YX, Zhang Z, Zhou XY, Zuo XL, Cong Y, Li YQ. Endomicroscopy will track injected mesenchymal stem cells in rat colitis models. *Inflamm Bowel Dis* (2015) doi:10.1097/MIB.0000000000000458
155. Lin Y, Lin L, Wang Q, Jin Y, Zhang Y, Cao Y, Zheng C. Transplantation of human umbilical mesenchymal stem cells attenuates dextran sulfate sodium-induced colitis in mice. *Clin Exp Pharmacol Physiol* (2015) doi:10.1111/1440-1681.12321
156. Liu W, Zhang S, Gu S, Sang L, Dai C. Mesenchymal stem cells recruit macrophages to alleviate experimental colitis through TGFβ1. *Cell Physiol Biochem* (2015) doi:10.1159/000369743
157. Onishi R, Ohnishi S, Higashi R, Watari M, Yamahara K, Okubo N, Nakagawa K, Katsurada T, Suda G, Natsuizaka M, et al. Human amnion-derived mesenchymal stem cell transplantation ameliorates dextran sulfate sodium-induced severe colitis in rats. *Cell Transplant* (2015) doi:10.3727/096368915X687570
158. Sun T, Gao GZ, Li RF, Li X, Li DW, Wu SS, Yeo AET, Jin B. Bone marrow-derived mesenchymal stem cell transplantation ameliorates oxidative stress and restores intestinal mucosal permeability in chemically induced colitis in mice. *Am J Transl Res* (2015)
159. Park JS, Yi TG, Park JM, Han YM, Kim JH, Shin DH, Tak SJ, Lee K, Lee YS, Jeon MS, et al. Therapeutic effects of mouse bone marrow-derived clonal mesenchymal stem cells in a mouse model of inflammatory bowel disease. *J Clin Biochem Nutr* (2015) doi:10.3164/jcbn.15-56

160. Wang X, Lazorchak AS, Song L, Li E, Zhang Z, Jiang B, Xu RH. Immune modulatory mesenchymal stem cells derived from human embryonic stem cells through a trophoblast-like stage. *Stem Cells* (2016) doi:10.1002/stem.2242
161. Mao F, Wu Y, Tang X, Wang J, Pan Z, Zhang P, Zhang B, Yan Y, Zhang X, Qian H, et al. Human umbilical cord mesenchymal stem cells alleviate inflammatory bowel disease through the regulation of 15-LOX-1 in macrophages. *Biotechnol Lett* (2017) doi:10.1007/s10529-017-2315-4
162. Yu Y, Song EM, Lee KE, Joo YH, Kim SE, Moon CM, Kim HY, Jung SA, Jo I. Therapeutic potential of tonsil-derived mesenchymal stem cells in dextran sulfate sodium-induced experimental murine colitis. *PLoS One* (2017) doi:10.1371/journal.pone.0183141
163. An JH, Song WJ, Li Q, Kim SM, Yang JI, Ryu MO, Nam AR, Bhang DH, Jung YC, Youn HY. Prostaglandin E 2 secreted from feline adipose tissue-derived mesenchymal stem cells alleviate DSS-induced colitis by increasing regulatory T cells in mice 11 Medical and Health Sciences 1107 Immunology. *BMC Vet Res* (2018) doi:10.1186/s12917-018-1684-9
164. de Aguiar CF, Castoldi A, Andrade-Oliveira V, Ignacio A, da Cunha FF, Felizardo RJF, Bassi ÊJ, Câmara NOS, de Almeida DC. Mesenchymal stromal cells modulate gut inflammation in experimental colitis. *Inflammopharmacology* (2018) doi:10.1007/s10787-017-0404-6
165. Jo H, Eom YW, Kim HS, Park HJ, Kim HM, Cho MY. Regulatory dendritic cells induced by mesenchymal stem cells ameliorate dextran sodium sulfate-induced chronic colitis in mice. *Gut Liver* (2018) doi:10.5009/gnl18072
166. Nikolic A, Simovic Markovic B, Gazdic M, Randall Harrell C, Fellabaum C, Jovicic N, Djonov V, Arsenijevic N, L Lukic M, Stojkovic M, et al. Intraperitoneal administration of mesenchymal stem cells ameliorates acute dextran sulfate sodium-induced colitis by suppressing dendritic cells. *Biomed Pharmacother* (2018) doi:10.1016/j.biopha.2018.02.060
167. Niu G chao, Liu L, Zheng L, Zhang H, Shih DQ, Zhang X. Mesenchymal stem cell transplantation improves chronic colitis-associated complications through inhibiting the activity of toll-like receptor-4 in mice. *BMC Gastroenterol* (2018) doi:10.1186/s12876-018-0850-7
168. Soontararak S, Chow L, Johnson V, Coy J, Wheat W, Regan D, Dow S. Mesenchymal Stem Cells (MSC) Derived from Induced Pluripotent Stem Cells (iPSC) Equivalent to Adipose-Derived MSC in Promoting Intestinal Healing and Microbiome Normalization in Mouse Inflammatory Bowel Disease Model. *Stem Cells Transl Med* (2018) doi:10.1002/sctm.17-0305
169. Xu X, Wang Y, Zhang B, Lan X, Lu S, Sun P, Li X, Shi G, Zhao Y, Han H, et al. Treatment of experimental colitis by endometrial regenerative cells through regulation of B lymphocytes in mice. *Stem Cell Res Ther* (2018) doi:10.1186/s13287-018-0874-5
170. Chang YL, Lo HY, Cheng SP, Chang KT, Lin XF, Lee SP, Hsieh MF, Chan CK. Therapeutic effects of a single injection of human umbilical mesenchymal stem cells on acute and chronic colitis in mice. *Sci Rep* (2019) doi:10.1038/s41598-019-41910-x
171. Kagia A, Tzetis M, Kanavakis E, Perrea D, Sfougataki I, Mertzanian A, Varela I, Dimopoulou A, Karagiannidou A, Goussetis E. Therapeutic Effects of Mesenchymal Stem Cells Derived From Bone Marrow, Umbilical Cord Blood, and Pluripotent Stem Cells in a Mouse Model of Chemically Induced Inflammatory Bowel Disease. Inflammation (2019) doi:10.1007/s10753-019-01033-x
172. Kang I, Lee BC, Lee JY, Kim JJ, Lee SE, Shin N, Choi SW, Kang KS. Interferon-γ-mediated secretion of tryptophanyl-tRNA synthetases has a role in protection of human umbilical cord blood-derived mesenchymal stem cells against experimental colitisW. *BMB Rep* (2019) doi:10.5483/BMBRep.2019.52.5.134
173. Pan X hua, Li Q qing, Zhu X qing, Li Z an, Cai X min, Pang R qing, Ruan G ping. Mechanism and therapeutic effect of umbilical cord mesenchymal stem cells in inflammatory bowel disease. *Sci Rep* (2019) doi:10.1038/s41598-019-54194-y
174. Li Y, Ma K, Zhang L, Xu H, Zhang N. Human Umbilical Cord Blood Derived-Mesenchymal Stem Cells Alleviate Dextran Sulfate Sodium-Induced Colitis by Increasing Regulatory T Cells in Mice. *Front Cell Dev Biol* (2020) doi:10.3389/fcell.2020.604021
175. Nishikawa T, Maeda K, Nakamura M, Yamamura T, Sawada T, Mizutani Y, Ito T, Ishikawa T, Furukawa K, Ohno E, et al. Filtrated Adipose Tissue-Derived Mesenchymal Stem Cell Lysate Ameliorates Experimental Acute Colitis in Mice. *Dig Dis Sci* (2021) doi:10.1007/s10620-020-06359-3
176. Wang G, Joel MDM, Yuan J, Wang J, Cai X, Ocansey DKW, Yan Y, Qian H, Zhang X, Xu W, et al. Human umbilical cord mesenchymal stem cells alleviate inflammatory bowel disease by inhibiting ERK phosphorylation in neutrophils. *Inflammopharmacology* (2020) doi:10.1007/s10787-019-00683-5
177. Hidalgo-Garcia L, Molina-Tijeras JA, Huertas-Peña F, Ruiz-Malagón AJ, Diez-Echave P, Vezza T,

Rodríguez-Sojo MJ, Morón R, Becerra-Massare P, Rodríguez-Nogales A, et al. Intestinal mesenchymal cells regulate immune responses and promote epithelial regeneration in vitro and in dextran sulfate sodium-induced experimental colitis in mice. *Acta Physiol* (2021) doi:10.1111/apha.13699
178. Liang L, Dong C, Chen X, Fang Z, Xu J, Liu M, Zhang X, Gu DS, Wang D, Du W, et al. Human umbilical cord mesenchymal stem cells ameliorate mice trinitrobenzene sulfonic acid (TNBS)-induced colitis. *Cell Transplant* (2011) doi:10.3727/096368910X557245
179. Parekkadan B, Upadhyay R, Dunham J, Iwamoto Y, Mizoguchi E, Mizoguchi A, Weissleder R, Yarmush ML. Bone marrow stromal cell transplants prevent experimental enterocolitis and require host CD11b+ splenocytes. *Gastroenterology* (2011) doi:10.1053/j.gastro.2010.10.013
180. Castelo-Branco MTL, Soares IDP, Lopes D V., Buongusto F, Martinusso CA, do Rosario A, Souza SAL, Gutfilen B, Fonseca LMB, Elia C, et al. Intraperitoneal but not intravenous cryopreserved mesenchymal stromal cells home to the inflamed colon and ameliorate experimental colitis. *PLoS One* (2012) doi:10.1371/journal.pone.0033360
181. Chen QQ, Yan L, Wang CZ, Wang WH, Shi H, Su B Bin, Zeng QH, Du HT, Wan J. Mesenchymal stem cells alleviate TNBS-induced colitis by modulating inflammatory and autoimmune responses. *World J Gastroenterol* (2013) doi:10.3748/wjg.v19.i29.4702
182. Zuo D, Liu X, Shou Z, Fan H, Tang Q, Duan X, Cao D, Zou Z, Zhang L. Study on the interactions between transplanted bone marrow-derived mesenchymal stem cells and regulatory T cells for the treatment of experimental colitis. *Int J Mol Med* (2013) doi:10.3892/ijmm.2013.1529
183. Stavely R, Robinson AM, Miller S, Boyd R, Sakkal S, Nurgali K. Human adult stem cells derived from adipose tissue and bone marrow attenuate enteric neuropathy in the guinea-pig model of acute colitis. *Stem Cell Res Ther* (2015) doi:10.1186/s13287-015-0231-x
184. Stavely R, Robinson AM, Miller S, Boyd R, Sakkal S, Nurgali K. Allogeneic Guinea pig mesenchymal stem cells ameliorate neurological changes in experimental colitis. *Stem Cell Res Ther* (2015) doi:10.1186/s13287-015-0254-3
185. Xing Y, Chen X, Cao Y, Huang J, Xie X, Wei Y. Expression of Wnt and Notch signaling pathways in inflammatory bowel disease treated with mesenchymal stem cell transplantation: Evaluation in a rat model. *Stem Cell Res Ther* (2015) doi:10.1186/s13287-015-0092-3
186. Zhang Y, Jin Y, Lin Y, Lin LJ, Cao Y, Wang DX, Zheng CQ. Adipose-Derived Mesenchymal Stem Cells Ameliorate Ulcerative Colitis Through MIR-1236 Negatively Regulating the Expression of Retinoid-Related Orphan Receptor Gamma. *DNA Cell Biol* (2015) doi:10.1089/dna.2015.2961
187. Zuo D, Tang Q, Fan H, Shou Z, Liu X, Cao D, Zou Z. Modulation of nuclear factor-κB-mediated pro-inflammatory response is associated with exogenous administration of bone marrow-derived mesenchymal stem cells for treatment of experimental colitis. *Mol Med Rep* (2015) doi:10.3892/mmr.2014.3038
188. Chao K, Zhang S, Qiu Y, Chen X, Zhang X, Cai C, Peng Y, Mao R, Pevsner-Fischer M, Ben-Horin S, et al. Human umbilical cord-derived mesenchymal stem cells protect against experimental colitis via CD5+ B regulatory cells. *Stem Cell Res Ther* (2016) doi:10.1186/s13287-016-0376-2
189. Robinson AM, Rahman AA, Miller S, Stavely R, Sakkal S, Nurgali K. The neuroprotective effects of human bone marrow mesenchymal stem cells are dose-dependent in TNBS colitis. *Stem Cell Res Ther* (2017) doi:10.1186/s13287-017-0540-3
190. Xie M, Qin H, Luo Q, He X, He X, Lan P, Lian L. Comparison of Adipose-Derived and Bone Marrow Mesenchymal Stromal Cells in a Murine Model of Crohn's Disease. *Dig Dis Sci* (2017) doi:10.1007/s10620-016-4166-6
191. de la Portilla F, Yuste Y, Pereira S, Olano C, Maestre MV, Padillo FJ. Local mesenchymal stem cell therapy in experimentally induced colitis in the rat. *Int J Stem Cells* (2018) doi:10.15283/ijsc17074
192. Fu ZW, Zhang ZY, Ge HY. Mesenteric injection of adipose-derived mesenchymal stem cells relieves experimentally-induced colitis in rats by regulating Th17/treg cell balance. *Am J Transl Res* (2018)
193. Martín Arranz E, Martín Arranz MD, Robredo T, Mancheño-Corvo P, Menta R, Alves FJ, Suárez De Parga JM, Mora Sanz P, De La Rosa O, Büscher D, et al. Endoscopic submucosal injection of adipose-derived mesenchymal stem cells ameliorates TNBS-induced colitis in rats and prevents stenosis. *Stem Cell Res Ther* (2018) doi:10.1186/s13287-018-0837-x
194. Guardia RDD La, Lopez-Millan B, Roca-Ho H, Bueno C, Gutiérrez-Agüera F, Fuster JL, Anguita E, Zanetti SR, Vives S, Nomdedeu J, et al. Bone marrow mesenchymal stem/stromal cells from risk-stratified acute myeloid leukemia patients are anti-inflammatory in in vivo preclinical models of

hematopoietic reconstitution and severe colitis. *Haematologica* (2019) doi:10.3324/haematol.2018.196568
195. Gao JG, Yu MS, Zhang MM, Gu XW, Ren Y, Zhou XX, Chen D, Yan TL, Li YM, Jin X. Adipose-derived mesenchymal stem cells alleviate TNBS-induced colitis in rats by influencing intestinal epithelial cell regeneration, Wnt signaling, and T cell immunity. *World J Gastroenterol* (2020) doi:10.3748/wjg.v26.i26.3750
196. Kuriyama T, Yamato M, Homma J, Tobe Y, Tokushige K. A novel rat model of inflammatory bowel disease developed using a device created with a 3D printer. *Regen Ther* (2020) doi:10.1016/j.reth.2019.12.005
197. Fawzy SA, Abo-Elnou RKE din, El-Deeb DFAEM, Abd-Elkader MMY. The possible role of mesenchymal stem cells therapy in the repair of experimentally induced colitis in male albino rats. *Int J Stem Cells* (2013) doi:10.15283/ijsc.2013.6.2.92
198. Mashhouri S, Froushani SMA, Tehrani AA. Non-adherent bone marrow-derived mesenchymal stem cells ameliorate clinical manifestations and inflammation in an experimental model of ulcerative colitis in rats. *Iran J Med Sci* (2020) doi:10.30476/ijms.2020.72514.0
199. Jung WY, Kang JH, Kim KG, Kim HS, Jang BI, Park YH, Song IH. Human adipose-derived stem cells attenuate inflammatory bowel disease in IL-10 knockout mice. Tissue Cell (2015) doi:10.1016/j.tice.2014.12.001
200. Pérez-Merino EM, Usón-Casaús JM, Zaragoza-Bayle C, Duque-Carrasco J, Mariñas-Pardo L, Hermida-Prieto M, Barrera-Chacón R, Gualtieri M. Safety and efficacy of allogeneic adipose tissue-derived mesenchymal stem cells for treatment of dogs with inflammatory bowel disease: Clinical and laboratory outcomes. *Vet J* (2015) doi:10.1016/j.tvjl.2015.08.003
201. Pérez-Merino EM, Usón-Casaús JM, Duque-Carrasco J, Zaragoza-Bayle C, Mariñas-Pardo L, Hermida-Prieto M, Vilafranca-Compte M, Barrera-Chacón R, Gualtieri M. Safety and efficacy of allogeneic adipose tissue-derived mesenchymal stem cells for treatment of dogs with inflammatory bowel disease: Endoscopic and histological outcomes. *Vet J* (2015) doi:10.1016/j.tvjl.2015.07.023
202. Ishioka S, Hosokawa T, Ikeda T, Konuma N, Kaneda H, Ohashi K, Furuya T, Masuko T, Taniguchi H, Kano K, et al. Therapeutic potential of mature adipocyte-derived dedifferentiated fat cells for inflammatory bowel disease. *Pediatr Surg Int* (2020) doi:10.1007/s00383-020-04681-5
203. Jung KJ, Lee GW, Park CH, Lee TJ, Kim JY, Sung EG, Kim SY, Jang BI, Song IH. Mesenchymal stem cells decrease oxidative stress in the bowels of interleukin-10 knockout mice. *Gut Liver* (2020) doi:10.5009/GNL18438
204. Nemoto Y, Kanai T, Takahara M, Oshima S, Nakamura T, Okamoto R, Tsuchiya K, Watanabe M. Bone marrow-mesenchymal stem cells are a major source of interleukin-7 and sustain colitis by forming the niche for colitogenic CD4 memory T cells. *Gut* (2013) doi:10.1136/gutjnl-2012-302029
205. Zhou C, Wu XR, Liu HS, Liu XH, Liu GH, Zheng X Bin, Hu T, Liang ZX, He XW, Wu XJ, et al. Immunomodulatory Effect of Urine-derived Stem Cells on Inflammatory Bowel Diseases via Downregulating Th1/Th17 Immune Responses in a PGE2-dependent Manner. *J Crohn's Colitis* (2020) doi:10.1093/ecco-jcc/jjz200
206. Duijvestein M, Vos ACW, Roelofs H, Wildenberg ME, Wendrich BB, Verspaget HW, Kooy-Winkelaar EMC, Koning F, Zwaginga JJ, Fidder HH, et al. Autologous bone marrow-derived mesenchymal stromal cell treatment for refractory luminal Crohn's disease: Results of a phase I study. *Gut* (2010) doi:10.1136/gut.2010.215152
207. Forbes GM, Sturm MJ, Leong RW, Sparrow MP, Segarajasingam D, Cummins AG, Phillips M, Herrmann RP. A phase 2 study of allogeneic mesenchymal stromal cells for luminal crohn's disease refractory to biologic therapy. *Clin Gastroenterol Hepatol* (2014) doi:10.1016/j.cgh.2013.06.021
208. Melmed GY, Pandak WM, Casey K, Abraham B, Valentine J, Schwartz D, Awais D, Bassan I, Lichtiger S, Sands B, et al. Human placenta-derived cells (PDA-001) for the treatment of moderate-to-severe Crohn's disease: A phase 1b/2a study. *Inflamm Bowel Dis* (2015) doi:10.1097/MIB.0000000000000441
209. Gregoire C, Briquet A, Pirenne C, Lechanteur C, Louis E, Beguin Y. Allogeneic mesenchymal stromal cells for refractory luminal Crohn's disease: A phase I–II study. *Dig Liver Dis* (2018) doi:10.1016/j.dld.2018.08.015
210. Zhang J, Lv S, Liu X, Song B, Shi L. Umbilical cord mesenchymal stem cell treatment for Crohn's disease: A randomized controlled clinical trial. *Gut Liver* (2018) doi:10.5009/gnl17035
