# Supplementary material for: Improving the Efficacy of Mesenchymal Stem/Stromal-Based Therapy for Treatment of Inflammatory Bowel Diseases
Source: Biomedicines. 2021 Oct 20;9(11):1507. doi: 10.3390/biomedicines9111507 (PMC8615066; doi:10.3390/biomedicines9111507)
Supplement: Supplementary file 1 [file biomedicines-09-01507-s001.zip › biomedicines-1399337-suppl 2/Supplementary Table S1_proofreading.pdf]

Supplementary Table S1. Characteristics of MSC-based therapy in DSS-induced colitis (listed in chronological order)

Reference	Animal model	Specie, Strain, Gender	MHC context	Source of MSCs	Administration route	MSC Dose	No. of MSCs infusions	MSCs used	Day of infusion (D)	Parameters analysed for IBD progression	Therapeutic effect of MSC therapy
Tanaka F, 2008 (142)	7-day DSS cycle	Rat, Lewis, M	S	BM	IV	$5x10^6$	x3	WT MSCs	D0, D2 & D4	-DAI (Body weight, stool consistency & food & water intake)<br>-Colon length & H/E staining<br>-COX2, bFGF, HGF, IL1β, TNFα & VEGF (colon)	Yes
Gonzalez-Rey E, 2009 (34)	-7-day DSS cycle<br>-2x7-day DSS cycle every 10 days	Mouse, C57BL6, NA	-S<br>-X<br>-A	AD	IP	$-1x10^5$<br>$-1x10^6$<br>$-5x10^6$	-x1<br>-x2	WT MSCs	-D-1<br>-D-7<br>-D7	-DAI (Body weight, stool consistency & rectal bleeding)<br>-Colon H/E staining	-Yes, D7<br>-Yes, A=S=X<br>-No, D-1 & D-7
Yabana T, 2009 (143)	-5-day DSS cycle<br>-7-day DSS cycle	Rat, Lewis & SD-TG, NA	S	BM	IP	NA	x1	WT MSCs	D7	-Body weight<br>-Colon length, H/E staining, IHC (Ki67) & tunel analysis<br>-Claudin 2, 12 & 15, IFNγ, IL1β, IL10 & TNFα (colon)	Yes
Zhang Q, 2009 (144)	7-day DSS cycle	Mouse, C57BL6, NA	X	-G<br>-BM	IP	$2x10^6$	x1	WT MSCs	D1	-DAI (Body weight, stool consistency & rectal bleeding)<br>-Colon H/E staining & MPO activity<br>-CD4, Foxp3, IFNγ, IL6, IL10 & IL17 (colon)	Yes
Tanaka H, 2011 (145)	6-day DSS cycle	Rat, Lewis, M	S	BM	IV	$2x10^4$	x1	WT MSCs	D2	-Body weight<br>-Colon H/E & alcian blue staining, IHC (CD31, CD45, PCNA, RM4 & SMA) & Tunnel analysis<br>-EGF, fgf2, Hes1, HGF, IFNγ, IGF1, IL1β, IL10, notch1Ptgs2, TFGα, TFGβ1, TNFα &VEGF (colon)	Yes
He XW, 2012 (146)	6-day DSS cycle	Mouse, BALBC, M	S	BM	IV	$1x10^6$	x3	WT MSCs	-D2, D5 & D8	-Body weight, stool consistency & rectal bleeding<br>-Colon length & H/E staining	Yes

Li L,<br>2013 (147)	7-day DSS cycle	Mouse,<br>C57/BL6,<br>F	X	-UC<br>-BM	IP	1x10^{6}	x1	WT MSCs	D2	-TNFα (serum)<br>-IL1β & TNFα (Colon)<br>-Body weight<br>-Colon H/E staining & MPO activity<br>-IL6, IL10, IL17 & TFGβ (colon)<br>-Th17 & Treg (mLN & SP)	Yes, UC-MSCs=BM-MSCs
Xu X,<br>2013 (148)	10-day DSS cycle	Mouse,<br>C57BL6/J,<br>F	S	G	IV	2x10^{5}	x1	WT MSCs	D3	-DAI (Body weight, stool consistency & rectal bleeding)<br>-Colon length & H/E & TB staining<br>-Apoptotic CD3, Th17 & Treg (PB)	Yes, N-GMSCs> GMSCs> M-GMSCs
Abdel-Salam AG,<br>2014 (149)	3x7-day DSS cycles, every 14 days	Mouse,<br>Albino,<br>F	S	BM	IP	5x10^{6}	x1	WT MSCs	D7	-Body weight & stool consistency<br>-Colon H/E staining<br>-ICAM-1, IFN-γ, IL-23 & TNF-α (colon)	Yes
Da costa Goncalves F,<br>2014 (150)	7-day DSS cycle	Mouse,<br>C57/BL6,<br>M	S	AD	-IP<br>-IV	1x10^{6}	x2	WT MSCs	D1 & D5	-DAI (Body weight, stool consistency & rectal bleeding)<br>-Colon length & weight & H/E staining & IHC (Tunnel in CD3 T cells)<br>- IFNγ, IL2, IL4, IL6, IL17 & TNFα (colon)	Yes, IV>IP
Gonzalez-Rey E,<br>2014 (151)	-TNBS<br>-4-day DSS cycle	-Mouse,<br>BALB/C,<br>M<br>-Mouse,<br>C57/BL6,<br>M	NA	NA	-IP<br>-IV<br>-SC	1x10^{7}	NA	WT MSCs	NA	-DAI (Body weight & stool consistency)	NA
Lv Y,<br>2014 (152)	7-day DSS cycle	Mouse,<br>BALBC,<br>M	X	Mens	IV	1x10^{6}	x4	WT MSCs	D2, D4, D6 & D8	-DAI (Body weight, stool consistency & rectal bleeding)<br>-Colon length, H/E staining, MPO activity & IHC (Mac-1)<br>-IL2, IL4, IL10 & TNFα (colon)<br>- CD3+CD25+, CD3+CD8+, IL2, MHCII+CD11c+, TNFα & Treg (SP)	Yes

Wang C, 2014 (54)	7-day DSS cycle	Mouse, C57BL6, M	S	BM	IV	$1x10^6$	1	WT MSCs<br>WT MSCs + SB431542	D8	-DAI (Body weight, stool consistency & rectal bleeding)<br>-Colon H/E staining<br>-CD206, F4/80, IFNγ, IL6, IL17, pSMAD, pTFGβ & TNFα (colon)	-Yes, WT MSCs<br>-No, WT MSCs+SB431542
Banerjee A, 2015 (153)	7-day DSS cycle	Mouse, NOD.CB$_{17}$-*Prkdc*^{scid}/JM, NA	X	UC	IV	$1x10^6$	x1	WT MSCs	D1	-DAI (Body weight, stool consistency & rectal bleeding)<br>-Colon length & H/E staining & IHC (human nuclei) & MPO & catalase activity<br>- BIP, MMP2, MMP9, PERK, PKR-like ER kinase (colon)	Yes
Li M, 2015 (154)	5-day DSS cycel	Rat, Sprague Dawley, M	S	BM	-IV<br>-IP	$-4x10^6$<br>$-5x10^6$	-x1<br>-x3	WT MSCs	-6 h<br>-D0, D2 & D4	-DAI (Body weight, stool consistency & rectal bleeding)<br>-IHC (GFP MSCs)	Yes
Lin Y, 2015 (155)	7-day DSS cycle	Mouse, C57/BL6, M	X	UC (WJ)	IV	$2x10^6$	x1	WT MSCs	D2	-DAI (Body weight, stool consistency & rectal bleeding)<br>-Colon length, H/E staining & IHC (COX2 & iNOS)<br>-IL1β, IFNγ, IL6 & TNFα (serum)<br>-Claudin-1, COX2, iNOS, occluding & ZO-1 (colon)	Yes
Liu W, 2015 (156)	7-day DSS cycle	Mouse, C57/BL6, M	S	BM	IV	$1x10^6$	x1	WT MSCs	D7	-DAI (Body weight, stool consistency, rectal bleeding & survival)<br>-Colon length & H/E staining<br>-IFNγ, IL6, IL17, TGFβ & TNFα (colon)	Yes
Onishi R, 2015 (157)	5-day DSS cycle	Rat, Sprague Dawley, M	X	Amniotic fluid	IV	$1x10^6$	x1	WT MSCs	D1	-DAI (Body weight, stool consistency & rectal bleeding)<br>-Colon length, H/E staining, MPO activity & IHC (CD3, CD68 & CD163)	Yes

Sun T,<br>2015 (158)	7-day DSS cycle	Mouse,<br>BALBC,<br>F	A	BM	IV	$2x10^5$	x1	WT MSCs	D7	-CXCL1, CXCL2, IFNγ, IL1β, IL6, MIF & MCP1 & TNFα (colon)<br>-MCP1 (serum)<br>-Colon H/E staining & IHC (E-cadherin)<br>-DAO & D-Lac (serum)<br>-MDA and SOD (colon)	Yes
Park JS,<br>2015 (159)	6-day DSS cycle	Mouse,<br>C57BL6,<br>M	S	BM	IV	$4x10^5$	x3	WT MSCs	D1, D3 & D5	-DAI (Body weight, stool consistency, rectal bleeding & survival)<br>-Colon length, H/E staining, IHC (F4/80, NIMP & TNFα) & cell death<br>-$CD4^+$, $CD8^+$, IFNγ, IL1β, IL4, IL6, IL10, IL17, IL22, IL23, TFGβ, Th1, Th2, Th17, TNFα & Treg (colon)	Yes
Lee HJ,<br>2016 (27)	3x4-day DSS cycle	Mouse,<br>C57/BL6,<br>F	S	BM	IV	$1x10^7$	x3	WT-MSCs	D1, D3 & D5	-Body weight<br>-Colon length & H/E staining<br>-IL10, TGFβ & TNFα (colon)	Yes
Wang M,<br>2016 (23)	5-day DSS cycle	Mouse,<br>C57BL6,<br>M	S	BM	-IP<br>-IV<br>-IR	$1x10^6$	x1	WT-MSCs	D5	-Body weight, stool consistency & rectal bleeding<br>-Colon length, MPO activity, H/E staining & IHC (collagen, Foxp3 & Ki67)<br>-IL10 & TNFα (colon)	Yes, IP>IR>IV
Wang X,<br>2016 (160)	6-day DSS cycle	Mouse,<br>NA,<br>NA	X	-BM<br>-E	IP	$1x10^6$	x2	WT-MSCs	D2 & D3	-Body weight, stool consistency & rectal bleeding<br>-Colon length & H/E staining	Yes, BM-MSCs=T-MSCs
Mao F,<br>2017b (161)	10-day DSS cycle	Mouse,<br>KM,<br>M	X	UC	IV	$1x10^6$	x3	WT MSCs	D3, D6 & D9	-Body weight<br>-Colon length, H/E staining & IHC (CD206 & PCNA)<br>-SP H/E staining & IHC (CD206)<br>-IL1β, IL6, IL10, IP10, 15lox STAT3 & TNFα (colon & SP)	Yes

Yu Y, 2017b (162)	5-day DSS cycle	Mouse, C57BL6, M	X	T	IP	$1x10^6$	-x2<br>-x4	WT-MSCs	-D6 & D16<br>-D6, D9, D12 & D16	-DAI (Body weight, stool consistency, rectal bleeding & survival)<br>-Colon length & H/E staining<br>-IL1β, IL6, IL10, IL11, IL13 IL17 & TNFα (colon)	Yes, 6, 9, 12 & 16> 6 & 16
An JH, 2018 (163)	7-day DSS cycle	Mouse, C57/BL6, M	X	AD	IP	$2x10^6$	x1	WT MSCs	D1	-DAI (Body weight, stool consistency, rectal bleeding & general activity)<br>-Colon length & H/E staining<br>- IFNγ, IL1β, IL4, IL6, IL10, TNFα& Treg (colon)	Yes
De Aguiar CF, 2018 (164)	7-day DSS cycle	Mouse, C57/BL6, M	S	AD	IP	$1x10^6$	x2	WT MSCs	D1 & D5	-DAI (Body weight, stool consistency, rectal bleeding & general activity)<br>-Colon length & H/E staining & IHC (CD11b)<br>-Arg1, IFNγ, IL6, IL10, MCP1, TNFα & ZO-1 (colon)<br>-DCs & Macrophages (colon & mLN)	Yes
Jo H, 2018 (165)	2x5-day DSS cycle	Mouse, C57/BL6, F	S	BM	IP	$1x10^6$	x2	-WT-MSCs<br>-MSCs-primed DCs<br>-DCs	D6 & D16	-Body weight & survival<br>-Colon length, H/E staining & IHC (Foxp3)<br>-Foxp3, IFNγ, IL6, IL10, STAT3, pSTAT3, TGFβ & TNFα (colon)	-Yes, MSCs pretreated DCs>WT MSCs<br>-No, DCs
Nikolic A, 2018 (166)	7-day DSS cycle	Mouse, C57BL/6, NA	S	BM	IP	$2x10^6$	x3	-WT MSCs<br>-MSCs-primed DCs<br>-DCs	-12h<br>-D1 & D2	-DAI (Body weight, stool consistency & rectal bleeding)<br>-Colon H/E staining<br>-Gal-3, HGF, IL-1β, IL-6, IL12 & PGE2 (serum)	Yes, WT MSCs & MSCs-primed DCs
Niu GC, 2018 (167)	6x5-day DSS cycle	Mouse, C57BL6, M	X	UC	IV	$1x10^6$	x1	WT MSCs	NA	-DAI (Body weight, stool consistency, rectal bleeding & survival)<br>-Colon length, H/E & MT staining & IHC (IFNγ, IL1β, IL17A, NFκB, TLR4, TNFα & TRAF6)	Yes

Soontararak S, 2018 (168)	DSS	Mouse, CD1, F	S	AD	IV	1x10^6	x3	-WT MSCs<br>-MSCs derived from iPSs	D10, 13 & 16	-ALB, ALT & AST (serum)<br>-IFNγ, IL1β, IL17A, NFκB, TLR4, TNFα & TRAF6 (liver)<br>-DAI (Body weight, stool consistency & rectal bleeding)<br>-Colon H/E staining & IF (CD31, KI67 & Lgr5)<br>-CD3, Cd4, CD11b, CD103, Foxp3 & F4/80 (colon, mLN & SP)	Yes, WT MSCs=MSCs from IPSs
Xu X, 2018 (169)	7-day DSS cycle	Mouse, BALB/C, M	X	Mens	IV	1x10^6	x3	WT MSCs	D2, D5 & D8	-DAI (Body weight, stool consistency, rectal bleeding & survival)<br>-Colon length & H/E staining<br>-IL6, IL10 & TNFα (colon)<br>- CD3, CD4, CD8, CD19, CD25, CD83, CD138, Foxp3, IFN-γ, IL10 & IL-17 (SP)<br>-Bregs (mLN, PC & SP)	Yes
Chang YL, 2019 (170)	6-day DSS cycle	Mouse, C57BL/6JNarl, M	X	UC	IP	2x10^6	x1	WT MSCs	D1	-DAI (Body weight, stool consistency, general activity & survival)<br>-Colon length & H/E staining<br>-Eotaxin, GRO-α, IL17A, MIP1α & MIP2 (serum)	Yes
Kagia A, 2019 (171)	3-day DSS cycle	Mouse, C57BL/6, F	X	-BM<br>-UC<br>-E<br>-iPS	IP	5x10^6	x1	WT MSCs	D3	-Body weight & survival<br>-Colon aspect & H/E staining	Yes, BM-MSCs & UC-MSCs> EC-MSCs & iPSC-MSCs
Kang I, 2019 (172))	7-day DSS cycle	Mouse, C57/BL6, M	X	UC	IP	NA	x1	WT MSCs	D1	-DAI (Body weight, stool consistency, rectal bleeding, general activity & survival)<br>-Colon length, H/E staining, MPO activity & IHC (ARS & WRS)<br>-IFNγ, IL6, IL12 & TNFα (serum)	Yes

Kawata Y, 2019 (32)	7-day DSS cycle	Mouse, C57/BL6, M	X	AD	-IP<br>-IV	1x10^6	x1	WT MSCs	-D3<br>-D11	-DAI (Body weight, stool consistency & rectal bleeding)<br>-Colon length, H/E staining & IHC (Treg)<br>- CD206, Fizz1, EGF, HGF, IL4, IL6, IL10, IL17, iNOS, PGE2, TFGβ, TNFα, TSG6, VEGF & Ym1 (colon)	-Yes, D3<br>-No, D11
Pan XH, 2019 (173)	5x4-day DSS cycle, every 3 days	Mouse, C57BL6, M	-S<br>-X	UC	-IP<br>-IV	-5x10^6<br>-1x10^7	x3	WT MSCs	D1, D3 & D5	-DAI (Body weight, stool consistency, rectal bleeding & survival)<br>-Colon length, H/E staining & IHC (CD4 & F4/80)<br>-IL6 & TNFα (serum)<br>-Claudin-1, IL6, LC3AB, occludin, VEGF & VEHFR1 (Colon)	-Yes, IP>IV<br>-Yes, X>S
Li Y, 2020b (174)	-7-day DSS cycle<br>-2x7-day DSS cycle	Mouse, C57/BL6, F	X	UC	IP	2x10^6	x2	WT MSCs	D2&D4	-DAI (Body weight, stool consistency, rectal bleeding & general activity)<br>-Colon H/E staining, MPO activity & IHC (CD4 & Foxp3)<br>-CD4 & Treg (colon, mLN & SP)	Yes
Lopez-Santalla M, 2020 (25)	2x7-day DSS cycle, every 3 months	-Mouse, C57BL6, M & F<br>-Mouse, RAG-1, M & F	-X<br>-S<br>-A	AD	IP	3-5x10^6	x1	WT MSCs	-D3<br>-D4<br>-D5<br>-D6	-DAI (Body weight, stool consistency, rectal bleeding & survival)<br>-Colon H/E staining<br>-Myeloid cells (BM, colon P, PB & SP)	-Yes, X=S=A<br>-Yes, D3=D4=D5=D6<br>-Yes, RAG-1>C57BL6<br>-Y, 1st=2nd DSS cycles
Nishikawa T, 2020 (175)	5-day DSS cycle	Mouse, C57BL/6, M	S	AD	IP	1x10^6	-x1<br>-x3	MSC lysates	-D2<br>-D2, D3 & D4	-DAI (Body weight & survival)<br>-Colon length, H/E staining & MPO activity<br>-Claudin 1 & 2, IFNγ, IL-1β, IL6, IL17, KC, iNOS, PAI-1, occluding & TNFα	Yes, MSC lysates

										(colon)	
Wang G, 2020 (176)	DSS	Mouse, C57BL6, NA	X	UC	IP	$3x10^6$	x3	WT MSCs	D3, D6 & D9	-DAI (Body weight, stool consistency & rectal bleeding)<br>-Colon & SP length & H/E staining<br>-Colon IHC (CCL2, CCL3, CD11b, CXCR4, FAS, ICAM1 & VEGF)<br>-CCL2, CCL3, CD11b, CXCR4, pERK, ERK, FAS, IVAM1, IL1β, IL6, IL8, IL10, pNFκB, TNFα & VEGF & (colon)	Yes
Hidalgo-García L, 2021 (177)	4-day DSS cycle	Mouse, C57/BL6, M	X	Intestinal	IP	$1x10^6$	x1	WT MSCs	D1	-DAI (Body weight, stool consistency & rectal bleeding)<br>-Colon length & weight, H/E staining, IHC (Villin &Zo-1) & TUNEL assay<br>-ArgI, ICAM, IL-6, IL-12, iNOS & TNF-α (Colon)	Yes

Therapeutic effect; > better than, =similar to; <less than
