# Supplementary material for: Improving the Efficacy of Mesenchymal Stem/Stromal-Based Therapy for Treatment of Inflammatory Bowel Diseases
Source: Biomedicines. 2021 Oct 20;9(11):1507. doi: 10.3390/biomedicines9111507 (PMC8615066; doi:10.3390/biomedicines9111507)
Supplement: Supplementary file 1 [file biomedicines-09-01507-s001.zip › biomedicines-1399337-suppl 2/Supplementary Table S2_proofreading.pdf]

Supplementary Table S2. Characteristics of MSC-based therapy in TNBS-induced colitis (listed in chronological order)

Reference	Animal model	Specie, Strain, Gender	MHC context	Source of MSCs	Administration route	MSC Dose	No. of MSCs infusions	MSCs used	Day of infusion (D)	Parameters analysed for IBD progression	Therapeutic effect of MSC therapy
Hayashi Y, 2008 (21)	TNBS	Rat, Sprague Dawley, M	S	BM	Submucosal	-1x10^6<br>-1x10^7	x1	WT MSCs	D0	-Body weight<br>-Colon H/E staining & IHC (Desmin, PKH26, Smad TGFβ, VEGF & Vimentin)	Yes
Liang L, 2011 (178)	TNBS	Mouse, BALBC, M	X	UC	IV	1x10^6	x2	WT MSCs	D0 & D1	-Body weight<br>-Colon H/E staining & MPO activity<br>-IFNγ, IL6, IL17 & IL23 (colon)<br>- IFNγ, IL17 & RORγT (serum)	Yes
Parekkadan B, 2011 (179)	TNBS	Mouse, C57BL6, M	S	BM	-IP<br>-IV	-1x10^6<br>-0.25x10^6	x1	-WT MSCs<br>-MSCs-pretreated CD11b	D0	Body weight, stool consistency, rectal bleeding & survival<br>-Colon weight, cellularity & H/E staining<br>-LN Treg, cellularity & weight	Yes, WT MSCs=MSC-pretreated CD11b
Castelo-Branco MT, 2012 (180)	TNBS	Rat, Wistar, M	S	-BM<br>-AD	IP	2x10^6	x1	WT MSCs	D4	-Body weight & survival<br>-Endoscopic score<br>-Colon H/E & TUNEL staining & collagen fibre density<br>- IL1β, IL10, TGFβ, TNFα & VEGF (colon)	Yes
Chen QQ, 2013 (181)	TNBS	Mouse, BALBC, F	S	BM	IV	1x10^6	x1	WT MSCs	48h	-DAI (Body weight, stool consistency & rectal bleeding)<br>-Colon length & H/E staining & IHC (Ki67 & Lgr5)<br>-IFNγ, IL2, IL4, IL6, IL10, IL17A & TNFα (serum)<br>-FOXP3, GATA3 IFNγ, IL2, IL4, IL6,	Yes

Zuo D,<br>2013 (182)	TNBS	Rat,<br>Sprague<br>Dawley,<br>F	S	BM	IV	$1x10^6$	x3	WT MSCs	D0, D3 & D7	IL10, IL17A, RORγ, T-bet, TGFβ & TNFα (colon)<br>-DAI (Body weight, stool consistency & rectal bleeding)<br>-Colon H/E staining<br>-TNFα (serum)<br>-NFκBp65 & TNFα (colon)	Yes
Gonzalez-Rey E,<br>2014 (151)	-TNBS<br>-4-day DSS	-Mouse,<br>BALB/C,<br>M<br>-Mouse,<br>C57/BL6,<br>M	NA	NA	-IP<br>-IV<br>-SC	$1x10^7$	NA	WT MSCs	NA	-DAI (Body weight & stool consistency)	NA
Stavely R,<br>2015a (183)	TNBS	Pig,<br>Hartley Guinea,<br>M & F	X	-AD<br>-BM	IR	$1x10^6$	x1	WT MSCs	3 h	-Body weight<br>-Colon H/E staining<br>-CD45, CGRPIR, ChAT-IR,nNOS-IR, iNOS-IR, TH-IR & VACh-IR neurons (myenteric plexus)	Yes, AD-MSCs=BM-MSCs
Stavely R,<br>2015b (184)	TNBS	Pig,<br>Hartley Guinea,<br>M & F	A	-AD<br>-BM	IR	$1x10^6$	x1	WT MSCs	3 h	-Body weight<br>-Colon H/E staining<br>-CD45, ChAT-IR nNOS-IR & iNOS-IR neurons (myenteric plexus)	Yes
Xing Y,<br>2015 (185)	TNBS	Rat,<br>Sprague<br>Dawley,<br>M	NA	NA	IV	$2x10^6$	x1	WT MSCs	24h	-Colon H/E staining<br>-APC, atolm1, β-catenin, ces3, Cyclin D cyp4f1, fabp1, fzd3, gcnt3, GSK-3β, ighg, igi, LOC366772, LOC679314, Mcpt1, Mcpt2, C-myc, olfm4, phosphol, pigz, s100g, slc7a15, , slc36a1, slc37a2, rnf125, TCF4, tmem35, Wnt3a, Wnt5, Wnt11 & zp2 (colon)	Yes
Zhang Y,<br>2015 (186)	TNBS	Rat,<br>Wistar,	S	AD	IV	$1x10^7$	x1	WT MSCs	24h	-Colon H/E staining, MDA & MPO activity & IHC (RORγT)	Yes

		M								-mir1236, RORγT & TNFα (colon)	
Zuo D,<br>2015 (187)	TNBS	Rat,<br>Sprague<br>Dawley,<br>F	S	BM	IV	$1x10^{6}$	x3	WT MSCs	D0, D3 & D7	-DAI (Body weight, stool consistency & rectal bleeding)<br>-Colon H/E staining<br>-Foxp3 & Treg (colon)	Yes
Chao K,<br>2016 (188)	TNBS	Mouse,<br>BALBC,<br>NA	X	UC	IP	$1x10^{6}$	x1	WT MSCs	2h	-DAI (Body weight, stool consistency & rectal bleeding)<br>-Colon length, H/E staining & MPO activity<br>-$CD5^{+}$ B cells, $CD8^{+}$ T cells, Th1, Th2, Th17 & Treg (mLN & SP)<br>-IL-6, IL10, IL-12, IL17, IL-21, IL-23, TGFβ & TNF-α (serum)	Yes
Lopez-Santalla M,<br>2017 (18)	TNBS	Mouse,<br>C57BL6,<br>M	X	AD	IL	$3.2x10^{5}$	x1	WT MSCs	1h	-Body weight<br>-Colon H/E staining	Yes
Robinson AM,<br>2017 (189)	TNBS	Pig,<br>Guinea,<br>M & F	X	BM	IR	-$1x10^{5}$<br>-$1x10^{6}$<br>-$3x10^{6}$	x1	WT MSCs	3 h	-Colon H/ staining & IHC (CD45, ChAT & nNOS myenteric neurons)	Yes, $3x10^{6}$= $1x10^{6}$>$1x10^{5}$
Xie M,<br>2017 (190)	TNBS	Mouse,<br>BALBC,<br>NA	S	-AD<br>-BM	IP	$1x10^{6}$	x1	WT MSCs	12h	-DAI (Body weight, stool consistency, rectal bleeding & survival)<br>-Colon length & weight, H/E staining & IHC (IL12, TNFα & VEGF)<br>-IL10, IL12, TNFα & VEGF (colon)	Yes, AD-MSCs=BM-MSCs
De la portilla,<br>2018 (191)	TNBS	Rat,<br>Wistar,<br>NA	A	AD	IR	$2x10^{6}$	x1	WT MSCs	D3	-DAI (Body weight, stool consistency & rectal bleeding)<br>-Colon H/E staining	Yes
Fu ZW,<br>2018 (192)	TNBS	Rat,<br>Sprague-<br>Dawley,<br>M	S	AD	IL	$2x10^{6}$	x1	WT MSCs	24 h	-DAI (Body weight, stool consistency & rectal bleeding)<br>-Colon length weight, H/E staining, MPO activity & IHC (Ki67)<br>-IL1β, TNFα & TSG-6 (colon & serum)	Yes

Lian L, 2018 (36)	TNBS	Mouse, BALBC, M	S	BM	IP	$1x10^6$	x1	WT MSCs	-24h before TNBS<br>-D7	-FOXP3, IL10, IL17A, RORγ, pSTAT3, pSTAT5 & TGFβ(colon)<br>-Body weight<br>-Colon length & H/E & Masson´s trichrome staining, IHC (Fibronectin, ColI, ColIII, IL1, IL6, IL10, IL13 & TFGβ) & IF (E-Cadherin & a-SMAD)<br>-pSmad1, pSmad3 & TFGβ (colon)	Yes, D7>24h before TNBS
Lopez-Santalla M, 2018 (22)	TNBS	Mouse, C57BL6, M	X	AD	IP	$3.2x10^5$	x1	WT MSCs	1h	-DAI (Body weight, stool consistency, rectal bleeding & survival)<br>-Colon H/E staining	Yes
Martin-Arranz E, 2018 (193)	TNBS	Rat, SD-OFA, M	X	AD	IR	$1x10^7$	x1	WT MSCs	D1	-Body weight<br>-Colon endoscopy, H/E staining & length<br>-IL10 & Treg (mLN)	Yes
Alves VBF, 2019 (26)	TNBS	Mouse, BALBC, F	X	AD	NA	$1x10^6$	x1	WT MSCs	24 h	-DAI (Body weight & survival)<br>-Colon H/E & PAS staining & EPO & MPO activity<br>-Leukocytes, eosinophils, neutrophils & basophils (PB)<br>-IL4 (colon)<br>-Th1, Th17 & Treg, (Colon, mLN & SP)<br>-IL1β, IL4, IL5, IL6 IL12, IL17, IL23 & TNFα (colon)	Yes
Diaz de la Guardia R, 2019 (194)	TNBS	Mouse, Bagg albino, M	X	BM	IP	$1x10^6$	1	-WT MSCs<br>-MSCs from AML patients	12h	-Body weight, stool consistency & survival<br>-Colon H/E staining & length<br>- IL1b, IL6, MIP2 & TNFα (serum)	Yes, WT MSCs=MSCs from AML patients

Gao JG, 2020 (195)	TNBS	Rat, Wistar, M	S	AD	IV	$1x10^7$	x1	WT MSCs	D8	-DAI (Body weight, stool consistency & rectal bleeding)<br>-Colon length & H/E staining<br>-pANCA & ASCA (serum)<br>-β-catenin, caspase 3, FOXP3, Fz3, Fz5, GATA3, IFNγ, IL2, IL4, IL10, IL17, IL23, IL13, Ror2, RORγT, Tbet, TGFβ, Th1, Th2, Th17, Treg, Wnt3a, Wnt5a, & TUNEL assay (colon)	Yes
Kuriyama T, 2020 (196)	TNBS	Rat, Sprague Dawley, M	S	AD	IR	$3x10^6$	x1	WT MSCs	24 h	-Ulcer area<br>-Colon H/E staining	Yes

Therapeutic effect; > better than, =similar to; <less than
