# Supplementary material for: Improving the Efficacy of Mesenchymal Stem/Stromal-Based Therapy for Treatment of Inflammatory Bowel Diseases
Source: Biomedicines. 2021 Oct 20;9(11):1507. doi: 10.3390/biomedicines9111507 (PMC8615066; doi:10.3390/biomedicines9111507)
Supplement: Supplementary file 1 [file biomedicines-09-01507-s001.zip › biomedicines-1399337-suppl 2/Supplementary Table S3_proofreading.pdf]

Supplementary Table S3. Characteristics of MSC-based therapy in less commonly used experimental models of IBD (listed in chronological order)

Reference	Animal model	Specie, Strain, Gender	MHC context	Source of MSCs	Administration route	MSC Dose	No. of MSCs infusions	MSCs used	Day of infusion (D)	Parameters analysed for IBD progression	Therapeutic effect of MSC therapy
Fawzy SA, 2013 (197)	Acetic acid	Rat, Albino, M	S	BM	IV	$5x10^5$	x1	WT MSCs	24h	-Colon H/E, Alcalin blue & Masson trichrome staining<br>-Collagen & mucin (colon)	Yes
Mashhouri S, 2020 (198)	Acetic acid	Rat, Wistar albino, M	S	BM	IP	$2x10^6$	x1	-Non-adherent MSCs<br>-Adherent MSCs	NA	-DAI (Stool consistency & rectal bleeding)<br>-Colon H/E staining, MPO & NO activities & lipid peroxidation<br>-IL1β, IL6 & TNFα (colon)	Yes, adherent MSCs= Non-adherent
Jung WY, 2015 (199)	7-day Piroxican cycle	Mouse, IL10 KO, NA	X	AD	IP	$2x10^6$	x1	WT MSCs	D7	-Body weight<br>-Colon length & H/E staining<br>-IFNγ, IL4, IL12, RANTES, TNFα & TLR9 (colon)	Yes
Perez-Merino EM, 2015a (200)	Idiopathic IBD	Dogs, NA, NA	A	AD	IV	$2x10^6$/Kg	x1	WT MSCs	Histological confirmation	-DAI (Body weight)<br>-Endoscopic analysis<br>-Albumin, cobalamin, CRP & folate CRP (serum)	Yes
Perez-Merino EM, 2015b (201)	Idiopathic IBD	Dogs, NA, NA	A	AD	IV	$2x10^6$/Kg	x1	WT MSCs	Histological confirmation	-Body weight<br>-DAI<br>-Albumin, cobalamin, folate & CRP (serum)	Yes
Ishioka S, 2020 (202)	Adoptive transfer	Mouse, SCID, NA	X	AD	IP	$1x10^5$	x1	WT MSCs	D7	-DAI (Body weight, stool consistency & rectal bleeding)	Yes
Jung KJ, 2020 (203)	IL10 KO	Mouse, IL10 KO, NA	X	BM	IV	$5x10^5$	x1	WT MSCs	1 week	-Colon length & H/E staining<br>-Colon macroscopic analysis<br>-Colon H/E & Masson´s trichrome staining<br>-CD4, CD8, Col II, IFNγ, IL4, pNFκB/p65 & TNFα (colon)<br>-Catalase, hydrogen peroxide, MDA, SOD1, SOD2 & Superoxide (Small intestine)	Yes

Therapeutic effect; > better than, =similar to; <less than
