# Supplementary material for: Improving the Efficacy of Mesenchymal Stem/Stromal-Based Therapy for Treatment of Inflammatory Bowel Diseases
Source: Biomedicines. 2021 Oct 20;9(11):1507. doi: 10.3390/biomedicines9111507 (PMC8615066; doi:10.3390/biomedicines9111507)
Supplement: Supplementary file 1 [file biomedicines-09-01507-s001.zip › biomedicines-1399337-suppl 2/Supplementary Table S4_proofreading.pdf]

Supplementary Table S4. Mechanisms involved in the therapeutic effects of MSC-based therapy using different molecule-silencing approaches (listed in chronological order)

Reference	Animal model	Specie, Strain, Gender	MHC context	Source of MSCs	Administration route	MSC Dose	No. of infusions	Molecule silenced in MSCs	Day of infusion (D)	Parameters analysed for IBD progression	Therapeutic effect of MSC therapy
Akiyama K, 2012 (38)	10-day DSS cycle	Mouse, C57BL6/J, F	A	BM	IV	$1x10^6$	1	-WT MSCs<br>-$FASL^{-/-}$ MSCs	D3	-DAI (Body weight, stool consistency & rectal bleeding)<br>-$AnxV^+7\text{-}AAD^+CD3^+$ cells (PB)<br>-Th17 & Treg (colon)	-Yes, WT MSCs<br>-No, $FASL^{-/-}$ MSCs
An JH, 2020b (43)	7-day DSS cycle	Mouse, C57/BL6, M	X	AD	IP	100 µg	3	-EVs from WT MSCs<br>-EVs from $TSG\text{-}6^{-/-}$ MSCs	D1, D3 & D5	-DAI (Body weight, stool consistency, rectal bleeding & general activity)<br>-Colon length & H/E staining<br>-Arg, CD11b, CD11c CD206, COX-2, FOXP3, IFNγ, IL6, IL10, IL17, iNOS, TGF-β, TNF-α & TSG-6, (colon)	-Yes, EVs from WT MSCs<br>-No, EVs from $TSG\text{-}6^{-/-}$ MSCs
Fan H, 2012 (44)	7-day DSS cycle	Mouse, C57/BL6, M	X	UC	IV	$2x10^6$	1	-WT MSCs<br>-IL1β-pretreated MSCs<br>-$IL1R1^{-/-}$ MSCs	D1	-Body weight, stool consistency & rectal bleeding<br>-Colon length, weight & H/E staining<br>-M1 & M2 macrophages (PC)<br>-Th1, Th2, Th17 & Treg (SP & mLNs)	Yes, IL1β-pretreated MSCs>$IL1R1^{-/-}$ MSCs>WT MSCs

Feng Y, 2018 (50)	TNBS	Mouse, BALB/C, M	A	BM	IP	$2x10^6$	1	-WT MSCs<br>-MGP$^{-/-}$ MSCs	12h	-Body weight, stool consistency & survival<br>-Colon length & H/E staining<br>-CD3, CD4, CD8, IFNγ, IL1β, IL6, IL10, IL17 & TNFα (colon)	Yes, WT MSCs>MGP$^{-/-}$ MSCs
Giri J, 2020 (51)	6-day DSS cycle	-Mouse, C57BL6, F<br>-Mouse, IL10 KO, F	S	BM	IP	$1x10^7$	2	-WT MSCs<br>-IFNγ-pretreated MSCs<br>-CCL2$^{-/-}$ MSCs	D2 & D4	-DAI (Body weight)<br>-Colon H/E staining	-Yes, IFNγ-pretreated MSCs>WT MSCs<br>-No, CCL2$^{-/-}$ MSCs
Kang J, 2019 (56)	10-day DSS cycle	Mouse, BALB/C, NA	X	UC	IP	$3x10^6$	3	-WT MSCs<br>-WT MSCs+miR148b-5p inhibitor<br>-WT MSCs+15-lox-1<br>-miR148b-5p-MSCs	D3, D6 & D9	-DAI (Body weight & stool consistency)<br>-Colon length, H/E staining & IHC (PCNA)<br>-caspase 3, IL1β, IL6, 15lox1, PCNA, & TNFα (colon)	-Yes, miR148b-5p-MSCs=15-lox-1-WT MSCs=WT MSCs<br>-No, WT MSCs+miR148b-5p inhibitor
Liao Y, 2016 (48)	TNBS	Mouse, C57/BL6, NA	S	BM	IP	$2x10^6$	1	-WT MSCs<br>-IGFBP7$^{-/-}$ MSCs	12 h	-Body weight & survival<br>-Colon length & H/E staining<br>- IFNγ & TNFα in CD4 & CD8 T cells (mLN)	-Yes, WT MSCs<br>-No, IGFBP7$^{-/-}$ MSCs
Nemoto Y, 2013 (204)	Adoptive transfer	-Mouse, RAG1 KO, NA<br>-Mouse, RAG1 & IL7 KO,	S	BM	IV	$1x10^6$	1	-WT MSCs<br>-IL7$^{-/-}$ MSCs	-D-28<br>-D0<br>-D1 & D28	-DAI (Body weight, stool consistency & rectal bleeding)<br>-Colon length & H/E staining<br>-IFNγ, IL17 & TNFαin	-Yes, WT MSCs<br>-No, IL7$^{-/-}$ MSCs

		NA<br>-mouse,<br>RAG2 KO,<br>NA								CD4 T cells (colon, mLN & SP)	
Parekkadan B,<br>2012 (47)	Adoptive transfer	Mouse,<br>RAG1 KO,<br>NA	S	BM	IV	$2x10^5$	2	-WT MSCs<br>-Aire$^{-/-}$ MSCs	-D0 & D21	-DAI (Body weight)<br>-Colon H/E staining<br>-TNFα (serum)<br>-IL6, IL10, Th1 Tr1, Treg & TNFα (colon)	-Yes, WT MSCs<br>-No, Aire$^{-/-}$ MSCs
Qiu Y,<br>2017 (46)	TNBS	Mouse,<br>BALBC,<br>NA	-X<br>-S	UC	IP	$1x10^6$	1	-WT MSCs<br>-Poly (I:C)-pretreated MSCs<br>-TNFα-pretreated MSCs<br>-IFNγ-pretreated MSCs<br>-LPS-pretreated MSCs<br>-WT MSCs+DAPT<br>-TLR3$^{-/-}$ MSCs<br>-Jagged-1$^{-/-}$ MSCs<br>-Notch-1$^{-/-}$ MSCs	-2 h<br>-D4	-DAI (Body weight, stool consistency & survival)<br>-Colon H/E staining & MPO activity<br>-COX2, IFNγ, IL4, IL6, IL10, IL17A, IL21, IL23, PGE2 & TNFα (colon & serum)<br>-Th1, Th17 & Treg (mLN & SP)	-Yes, Poly (I:C)-pretreated MSCs>IFNγ-pretreated MSCs>LPS-pretreated MSCs=TNFα-pretreated MSCs=WT MSCs<br>-Yes, S=X<br>-Yes, 12h & D4<br>-No, WT-MSCs+DAPT, TLR3$^{-/-}$ MSCs,Jagged-1$^{-/-}$ MSCs & Notch-1$^{-/-}$ MSCs
Sala E,<br>2015 (20)	-10-day DSS cycle<br>-TNBS	-Mouse,<br>C57BL6,<br>F<br>-Mouse,	-S<br>-A	-BM<br>-AD	-IV<br>-IP<br>-SC	$3x10^6$	-5<br>-4	-WT MSCs<br>-TSG6$^{-/-}$ MSCs<br>-Encapsulated MSCs<br>-TSG-6	-D5-D9<br>-D1-D4	-DAI (Body weight, stool consistency & rectal bleeding)<br>-Colon length & H/E	-Yes, TSGW>TMSCs=encapsulated MSCs<br>-No, TSG6$^{-/-}$

		BALB/C, F								staining<br>-Arg-II, B220^{+}, CCL22, CD3^{+}, CD11b^{+}F4/80^{+}, CD11^{+}Ly6G$^{+,}$ Foxp3, HEMO-1, IL10, IL12, TGF-β & TNFα (colon & P)<br>-TSG-6 (serum)	MSCs
Song WJ, 2017 (42)	7-day DSS cycle	Mouse, C57BL6, M	X	AD	IP	2x10^{6}	1	-WT MSCs<br>-TSG6$^{-/-}$ MSCs	D1	-DAI (Body weight, stool consistency & rectal bleeding)<br>-Colon H/E staining & IHC (CD11b & CD206)<br>-Arg1, CD206, Fizz1, IFNγ, IL1β IL10, IL17, TNFα & Ym1 (colon)	-Yes, WT MSCs<br>-No, TSG6$^{-/-}$ MSCs
Song WJ, 2018 (41)	7-day DSS cycle	Mouse, C57BL6, M	X	AD	IP	2x10^{6}	-1	-WT MSCs<br>-TSG6$^{-/-}$ MSCs	D1	-DAI (Body weight, stool consistency & rectal bleeding)<br>-Colon H/E staining & IHC (AnxV, CD11b, CD206 & PI)<br>-IL6, IL10 & TNFα (colon)	-Yes, WT MSCs<br>-No, TSG6$^{-/-}$ MSCs
Takeyama H, 2017 (49)	DSS	Mouse, C57BL6, M	S	AD	IP	2x10^{6}	1	-WT MSCs<br>-TSP-1$^{-/-}$ MSCs	D2	-Body weight<br>-Colon length & H/E staining<br>-colon, mLN, & SP IHC (GFP)<br>-CD4, CD11c, CD51, CD103 & Foxp3 (colon, mLN & SP)	-Yes, WT MSCs<br>-No, TSP-1$^{-/-}$ MSCs

Tian J, 2020 (57)	10-day DSS cycle	Mouse, C57BL6, NA	S	BM	IV	0.5 x 10^6	1	-WT MSCs<br>-Pitsopt2-pretreated MSCs<br>-Colivelin-pretreated MSCs<br>-siRNA-BECN1-MSCs	D3	-DAI (Body weight, stool consistency & rectal bleeding)<br>-Colon length & H/E staining<br>-Th17 & Treg (SP)<br>-BECN1, Corticosterone, IFNγ, CD4, CD8, CD9, CD63, CD81, LC3-I, LC3-II, pSTAT3, STAT3 & TNFα (PB)	-Yes, WT MSCs>colivelin-pretreated MSCS<br>-No, Pitsopt2-pretreated MSCs & siRNA-BECN1-MSCs
Wang C, 2014 (54)	7-day DSS cycle	Mouse, C57BL6, M	S	BM	IV	1x10^6	1	WT MSCs<br>WT MSCs+SB431542	D8	-DAI (Body weight, stool consistency & rectal bleeding)<br>-Colon H/E staining<br>-CD206, F4/80 IFNγ, IL6, IL17, pSMAD, pTFGβ & TNFα (colon)	-Yes, WT MSCs<br>-No, WT MSCs+SB431542
Wu T, 2015 (138)	9-day DSS cycle	Mouse, C57BL6, M	X	BM	IV	1x10^6	1	-WT MSCs<br>-mir21-/- MSCs	D3	-DAI (Body weight)<br>-Colon length & H/E staining<br>-Treg (colon)	Yes, Mir21-/-MSCs<br>> WT MSCs
Xu J, 2020 (55)	3x5-day DSS cycle every 10 days	Mouse, C57BL6, M	X	E	IV	5x10^5	3	-WT MSCs<br>-WT MSCs+OSI906<br>-WT MSCs+PPP<br>-IGF-1	D3, D18 &D33	-DAI (Body weight, stool consistency & rectal bleeding)<br>-Colon H/E staining & IHC (Ki67)<br>-Liver IF (F4/80)<br>-Lung IF (CD31)<br>-ACE, Adiponectin, BLC, CXCL1, CXCL2, IGF1, Igfbp 1, 2 3 & 4, IL5, IL6,	-Yes, WT MSCs>IGF-1<br>-No, WT MSCs+OSI906 & WT MSCs+PPP

										IL17BR, IL17F, ITAC, MCP1, PDGFaa, pentraxin3, SDF & e-selectin (serum)<br>- AKT, pAKT, PI3K IGF-1R, pIGF-1R β, p44/42 MAPK, mTOR, pmTOR, & p70s6 Kinase (colon)<br>-RNA sequencing analysis (colon)	
Yang R, 2018 (45)	10-day DSS cycle	Mouse, C57BL6, NA	S	G	IV	$0.2x10^{6}$	1	-WT MSCs<br>-NaHS-pretreated $Cbs^{-/-}$ MSCs<br>-$Cbs^{-/-}$ MSCs	D3	-DAI (Body weight & stool consistency)<br>-Colon H/E staining<br>-Th17 & Treg (colon)	-Yes, WT MSCs=NaHS-pretreated $Cbs^{-/-}$ MSCs<br>-No, $Cbs^{-/-}$ MSCs
Yang S, 2021 (39)	-7-day DSS cycle<br>-TNBS	-Mouse, C57BL6, M<br>-Mouse, BALB/C, M	X	UC	IP	200 µg	1	-EVs from WT MSCs<br>-siTSG-6 MSCs-EVs<br>-rhTSG-6	-D5<br>-24h	-DAI (Body weight, stool consistency, rectal bleeding & survival)<br>-Colon length, FITC, H/E & PAS staining, MPO activity & IHC (Claudin-1, Occludin & ZO-1)<br>-Claudin-1, CXCL14, IL-1β, IL-4, IL-11, IL-12 Occludin, TGF-β, TSG6 & ZO-1 (colon)<br>-Th2 & Th17 (mLN & SP)	-Yes, EVs from WT MSCs=TSG6<br>-No, siTSG-6 MSCs-EVs
Zhang S, 2021 (40)	7-day DSS cycle	Mouse, C57BL6, NA	X	Musc	IV	$2.5x10^{5}$	1	-WT MSCs<br>-IFN-γ+TNFα-pretreated MSCs<br>-$IDO^{-/-}$ MSCs	D2	-Body weight<br>-Colon H/E staining<br>-IL6 (serum)	Yes, WT MSCs=TSG6>$IDO^{-/-}$ MSCs+TSG6=I

								-TSG6$^{-/-}$ MSCs<br>-TSG6<br>- IDO$^{-/-}$ MSCs+KYN<br>- IDO$^{-/-}$ MSCs+KYNA			DO$^{-/-}$ MSCs+KYN=IDO$^{-/-}$ MSCs+KYNA>IDO$^{-/-}$ MSCs=TSG6$^{-/-}$ MSCs
Zhou C, 2020 (205)	-2x7-day DSS cycle<br>-TNBS	-Mouse, C57BL/6, F<br>-Mouse, BALB/C, M	X	-U<br>-BM	IP	$1x10^6$	-1<br>-2	-WT MSCs<br>-siCOX2-MSCs	-D1<br>-D7<br>-D7 & D16	-DAI (Body weight, stool consistency, rectal bleeding & survival)<br>-Colon length, H/E & Masson´s trichrome staining, MPO activity & IHC (IFN-γ, IL-10 & IL-17)<br>- GCSF, IFN-γ, IL1β, IL2, IL3, IL6, IL7, IL-10, IL12p70, IL15, IL-17, IL23, PGE2 & TNFα (colon)<br>-Th1, Th17, Tr1 & Treg (mLN)<br>-PGE2 (PB)	-Yes, BM-MSCs=U-MSCs<br>-Yes, WT MSCs>siCOX2-MSCs<br>-Yes, D7&D16>D7

Therapeutic effect; > better than, =similar to; <less than
