# Supplementary material for: Improving the Efficacy of Mesenchymal Stem/Stromal-Based Therapy for Treatment of Inflammatory Bowel Diseases
Source: Biomedicines. 2021 Oct 20;9(11):1507. doi: 10.3390/biomedicines9111507 (PMC8615066; doi:10.3390/biomedicines9111507)
Supplement: Supplementary file 1 [file biomedicines-09-01507-s001.zip › biomedicines-1399337-suppl 2/Supplementary Table S5_proofreading.pdf]

Supplementary Table S5. Completed clinical trials in IBD with systemic MSC-based therapy (listed in chronological order)

Clinical Trial Identifier	Clinical Phase	Source	Registration year	Country	IBD patients	MHC context	Route of administrati on	Cells/kg of Body Weight; Number of Doses	Number of IBD Patients Enrolled	Follow-Up (Months)	Publication (Year)	Included Control Group	Age range (years)
NCT00294112	II	BM	2006	USA	Refractory CD patients	Allogeneic	IV	-2x10^6/Kg, 2 doses =4 x 10^6<br>-8x10^6/Kg, 2 doses=16 x 10^6 weekly	10	1 month	Not published	No	18-70
NCT00543374	III	BM	2007	USA	Refractory CD patients	Allogeneic	IV	-6 x 10^8 cells, in 4 doses in two weeks<br>-12 x 10^8 cell in 4 doses in two weeks	98	6 months	Not published	Yes	18-70
NCT00482092	III	BM	2007	USA	Refractory CD patients	Allogeneic	IV	-6 x 10^8 cells, in 4 doses in two weeks<br>-12 x 10^8 cell in 4 doses in two weeks	330	1 month	Not published	Yes	18-70
NTR1360	I	BM	2008	The Netherlands	Refractory CD patients	Autologous	IV	1-2x10^6/Kg, 2 doses over two weeks= 2-4x10^6/Kg	10	3.5 months	2010 (206)	No	1-42
NCT01233960	III	BM	2010	USA	Refractory CD patients	Allogeneic	IV	-2x10^8, 12 doses= 24x$10^{8,}$ every 6 weeks	73	6 months	Not published	No	18-70
NCT01090817	II	BM	2010	Australia	Refractory CD patients	Allogeneic	IV	2x10^6/Kg, 4 doses weekly=8x10^6	21	1.5 months	2014 (207)	No	18-55
NCT01155362	IIa	Placenta	2010	USA	Refractory CD patients	Allogeneic	IV	-1.5x10^8, 2 doses=3x10^8<br>-6x10^8, 2 doses, 12x10^8<br>-12x10^8, 2 doses=24x10^8	50	1.5 months	2015 (208)	Yes	18-75
NCT01221428	I/II	UC	2010	China	ASA-treated moderate to severe UC patients	Allogeneic	IV + IA	2.3–4.7×10^7IV & 1.5×10^7IA one week later = 40x10^7	80	2 years	2016 (17)	Yes	18-70
NCT01540292	I/II	BM	2012	Belgium	Refractory CD patients	Allogeneic	IV	1.5-2x10^6/Kg, 2 doses=3-4x10^6/Kg	13	12 months	2018 (209)	No	18-75
NCT01659762	I	BM	2012	USA	Refractory CD patients	Autologous	IV	-2x10^6/Kg, 1 dose<br>-5x10^6/Kg, 1 dose<br>-10x10^6/Kg, 1 dose	16	3 months	2016 (65)	No	18-65
NCT02150551	Pilot/I Phase	BM	2015	USA	Refractory CD or UC paediatric patients	Allogeneic	IV	1x10^6 /Kg, 4 doses weekly=4x10^6/Kg	1	24 months	Not published	No	12-22
NCT02445547	I/II	UC	2015	China	CD patients	Allogeneic	IV	1x10^6/Kg, 4 doses= 4x10^6/Kg	41	12 months	2018 (210)	No	18-70
