# Supplementary material for: Improving the Efficacy of Mesenchymal Stem/Stromal-Based Therapy for Treatment of Inflammatory Bowel Diseases
Source: Biomedicines. 2021 Oct 20;9(11):1507. doi: 10.3390/biomedicines9111507 (PMC8615066; doi:10.3390/biomedicines9111507)
Supplement: Supplementary file 1 [file biomedicines-09-01507-s001.zip › biomedicines-1399337-suppl 2/Supplementary Table S6_proofreading.pdf]

Supplementary Table S6. Ongoing clinical trials in IBD with systemic MSC-based therapy (listed in chronological order)

Clinical Trial Identifier	Clinical Phase	Source	Registration year	Country	IBD patients	MHC context	Route of administration	Cells/kg of Body Weight; Number of Doses	Number of IBD Patients Enrolled	Follow-Up (Months)	Publication (Year)	Status	Included Control Group	Estimation date for completion	Age range (years)
2011-005966-39	II	NA	2012	Belgium	Refractory CD patients	NA	IV	NA	15				No		18-75
NCT01510431	NA	BM	2012	USA	Refractory CD patients	Allogeneic	IV	-2x10^{8}, 4 doses= 8x10^{8} every 3 days	NA	NA		No longer available	No	2020	18-70
NCT01914887	I/IIa	AD	2013	Spain	Early diagnosed and 5-ASA-resistant CU patients	Allogeneic	Local	60x10^{6}, 1 dose	8	3 months		Unknown status	No	Unknown status	>18
NCT02000362	I/IIa	UC	2014	Korea	Refractory CD patients	Allogeneic	IV	-5x10^{7}, 1 dose<br>-1x10^{8}, 1 dose	24	1 month		Recruiting	No	Unknown	19-70
NCT02442037	I/II	UC	2015	China	UC patients	Allogeneic	IV	1x10^{6}/Kg, 3 doses= 3x10^{6}/Kg	30	6 months		Recruiting	Yes	Unknown	18-65
NCT02580617	I	AD	2015	Korea	Refractory CD patients	Allogeneic	IV	-5x10^{7}, 1 dose<br>-7.5x10^{7}, 1 dose<br>-10x10^{7}, 1 dose	9	1 month		Recruiting	No	2021	18-65
NCT03183661	I	AD	2016	Korea	CD patients	Allogeneic	IV	NA	9	36 months		Enrolling by invitation	No	2021	18-65
NCT02926300	I/IIa	UC	2016	Korea	Refractory CD patients	Allogeneic	IV	NA	24	28 months		Recruiting	No	2021	19-70
NCT03299413	I/II	Wharton Jelly	2017	Jordan	Early diagnosed UC patients	Allogeneic	IV	60x10^{6}, 2 doses=120x10^{6}	20	6 months		Active, not recruiting	No	2019	18-75
NCT03609905	I/II	AD	2018	China	Early diagnosed and 5-ASA, glucocorticoid or azathioprine-resistant CU patients	Allogeneic	Local	5x10^{7}, 2 doses= 10x10^{7}	50	2 months		Recruiting	No	Unknown	18-65
NCT04548583	Ib/IIa	BM	2020	USA	Refractory CD patients	Allogeneic	Local	-150x10^{6}, 1 dose<br>-300x10^{6}, 1 dose	24	24 months		Recruiting	Yes	2023	18-75
NCT04312113	I	AD	2020	USA	Refractory UC patients	Autologous	IA	-15x10^{6}, 1 dose<br>-30x10^{6}, 1 dose	20	24 months		Recruiting	No	2022	18-65
NCT04543994	I/II	BM	2020	USA	Refractory UC patients	Allogeneic	Local	-150x10^{6}, 2 doses=300x10^{6}<br>-300x10^{6}, 2 doses=600x10^{6}	24	24 months		Recruiting	Yes	2023	18-75
